# Adverse Events of Concurrent Immune Checkpoint Inhibitors and Antiangiogenic Agents: A Systematic Review

**DOI:** 10.3389/fphar.2019.01173

**Published:** 2019-10-17

**Authors:** Ling Gao, Xi Yang, Cheng Yi, Hong Zhu

**Affiliations:** Department of Medical Oncology, Cancer Center, West China Hospital, Sichuan University, Chengdu, China

**Keywords:** immune checkpoint inhibitor, antiangiogenic monoclonal antibody, tyrosine kinase inhibitor, concurrent therapy, treatment-related adverse event, immune-related adverse event, systematic review

## Abstract

**Background:** Immune checkpoint blockade has revolutionized the treatment of multiple malignancies. Currently, however, the effect is not universal, with objective response rates (ORR) of about 15–25%, and even lower for some cancers. Abnormal vasculature is a hallmark of most solid tumors and plays a role in immune evasion. Growing body of evidence suggests that vascular normalization and immune reprogramming could operate synergistic effect, resulting in an enhanced therapeutic efficacy. However, the benefit of antitumor efficacy must be weighed against the risk of added toxicity. In this systematic review, we summarize severe toxicity observed in such a kind of combination regimen.

**Methods:** PubMed and Embase were searched for English references published up to May 31, 2019, with MeSH and keywords search terms of immune checkpoint inhibitors (ICIs) and antiangiogenic agents approved for using in solid tumors. Studies performing concomitant use of ICIs and antiangiogenic agents, and also reporting severe treatment-related adverse events (trAEs) (≥grade 3), were included for further analysis.

**Results:** A total of 32 studies including a total of 2,324 participants were analyzed. Limited available data suggests that both antiangiogenic monoclonal antibodies (mAbs) and tyrosine kinase inhibitors (TKIs) show potential risk of increasing treatment-related toxicity when combined with ICIs. Overall, the total incidence of severe adverse events (AEs) associated with ICIs plus mAbs (44.5%) is lower than that of ICIs plus TKIs (60.1%). However, the trAEs observed in combination therapy are mostly consistent with the known safety profiles of corresponding monotherapy, and they seem to be largely related to antiangiogenic agents, rather than a true immune-related adverse event (irAE) predominantly due to ICIs. The majority of trAEs are intervened by holding ICI treatment and adding corticosteroids, as well as reducing dose or adjusting administration frequency of the antiangiogenic drugs.

**Conclusions:** Concurrent use of ICIs and antiangiogenic agents shows potential treatment-related toxicity. Further research is required to compare the efficacy and safety of the combination regimen and corresponding monotherapy and identify predictive biomarkers, as well as explore dose, duration, and sequencing schedules of drugs.

## Introduction

Interventions for local advanced or metastatic solid tumors have evolved rapidly in recent years, among which immune checkpoint blockade therapy may be the most notable strategy (Pardoll, 2012; Hoos, 2016; Papaioannou et al., 2016). Indeed, immune checkpoint inhibitors (ICIs) targeting the cytotoxic T lymphocyte-associated protein 4 (CTLA-4) and programmed cell death protein 1 (PD-1), a T-cell immune checkpoint receptor, or its ligand PD-L1 may be effective for various types of cancer and have brought significant improvements in clinical prognosis (Hodi et al., 2010; Herbst et al., 2014; Ansell et al., 2015; Sharma and Allison, 2015). However, these therapies benefit just a few of patients, with objective response rates (ORR) of about 15–25%, and even lower for pancreatic carcinoma, prostate cancer, ovarian carcinoma, triple negative breast cancer, and microsatellite stable colorectal cancer. It may be attributed to insufficient abundance of tumor neoantigens, tumor heterogeneity, and genetic variation among individuals. Besides, acquired tumor resistance of ICIs is also a challenge (Ma et al., 2016; Wang and Wu, 2017). Therefore, it is necessary to seek combination therapy strategy which can activate anti-tumor immunity and enhance treatment efficacy.

Researches have identified that abnormal tumor vasculature in the tumor microenvironment (TME) not only fuels tumor progression but also has a negative impact on the effectiveness of all types of anticancer therapies, especially immunotherapy. Elevated interstitial fluid pressure of the TME caused by the leaky nature of tumor vessels and dysfunctional lymphatic drainage, along with low expression level of cell adhesion molecules, such as vascular cell adhesion protein 1 (VCAM1) and intercellular adhesion molecule 1 (ICAM1), limits the entry of drugs and the trafficking of immune effector cells into tumors (Griffioen et al., 1996; Buckanovich et al., 2008; Jain, 2013). Besides, angiogenic molecules presenting in the TME, such as vascular endothelial growth factor (VEGF), act as a mediator of tumor-associated immunosuppression. Firstly, VEGF directly prevent mobilization, trafficking, development, proliferation, and effector function of CD8-positive cytotoxic T lymphocytes (CTLs) (Ohm and Carbone, 2001; Voron et al., 2015). Secondly, VEGF could promote the recruitment and proliferation of immunosuppressive cells, including regulatory T (Treg) cells, myeloid-derived suppressor cells (MDSCs), and M2-like tumor-associated macrophages (TAMs) (Terme et al., 2013; Chaudhary et al., 2014; Maenhout et al., 2014). Thirdly, maturation and antigen presentation of dendritic cells (DCs) might be suppressed by elevated VEGF (Gabrilovich et al., 1996; Gabrilovich et al., 1998). Thus, strategies inducing vascular normalization may restore immune cell functions and help to attenuate the immunosuppression of the TME, thereby improve the activity of immunotherapy. For example, sunitinib could increase T-cell and B-cell levels and decrease PD-1 expression in tumor-infiltrating T-cells as well as inhibit MDSCs and Treg cells into tumor (Heine et al., 2011; Voron et al., 2015). Bevacizumab and pazopanib could increase the infiltration or activity of CD8-positive and CD4-positive T-cells and enhance the maturation of DCs (Elamin et al., 2015; Zizzari et al., 2018). However, recent studies have also shown that an adaptive immunosuppression caused by the up-regulation of PD-L1 in endothelial cells (ECs) and tumor cells after antiangiogenic therapies limits the activity of antiangiogenesis (Allen et al., 2017). It suggests that combination of antiangiogenesis and immune checkpoint blockade targeting PD-1/PD-L1 may be a good choice. More interestingly, bioinformatic analyses revealed that gene expression features related to vascular normalization correlate with immunostimulatory pathways, especially the activation and infiltration of T-cells. As a result, activating of CD4-positive T-cells by ICIs promoted the normalization of tumor vessels in return (Tian et al., 2017). Therefore, it demonstrates that vascular normalization and immune reprogramming have synergistic effect, which provides a basis for the rationality of the combination of ICIs and antiangiogenic agents.

Indeed, preclinical evidences have confirmed the efficacy of these combination regimens (Yasuda et al., 2013; Motoshima et al., 2015; Du Four et al., 2016; Kimura et al., 2018; Laubli et al., 2018). For instance, in a mouse model of colon adenocarcinoma, treatment with axitinib led to an improved T-cell response, and it resulted in a synergistic therapeutic efficacy when combined with anti-PD-1 antibody (Laubli et al., 2018). On the basis of preclinical data, these combination therapies have been tested in dozens of clinical trials, which reported promising outcomes in patients with metastatic melanoma, non-squamous non-small-cell lung carcinoma (NSCLC), and renal cell carcinoma (RCC). Among them, IMpower150 trial showed that atezolizumab plus chemotherapy plus bevacizumab significantly improved progression-free survival (PFS) and overall survival (OS) of patients with metastatic non-squamous NSCLC, regardless of mutational status and checkpoint expression of tumor (Reck et al., 2019). Similarly, in other two phase 3 trials on the first-line treatment of advanced or metastatic RCC, concomitant use of pembrolizumab and axitinib improved OS, PFS, and ORR over the standard of care (Rini et al., 2019a), while combining avelumab with axitinib improved PFS and ORR (Motzer et al., 2019).

However, despite the enhanced anti-tumor efficacy, the combination treatment is not without challenge, including the risk of added toxicity and increasing of immune-related adverse events (irAEs). As is well known, toxic effects associated with ICIs manifesting with autoimmune-like side-effects are commonly seen in the skin, gastrointestinal tract, pulmonary, hepatic, renal, nervous, hematologic, cardiovascular, and endocrine systems (Gordon et al., 2017; Puzanov et al., 2017). Likewise, antiangiogenic monoclonal antibodies (mAbs) and small-molecule tyrosine kinase inhibitors (TKIs), the two main types of antiangiogenic agent, also have diverse adverse effects, mainly including hypertension, arterial thromboembolic events, proteinuria, bowel perforation, reversible posterior leukoencephalopathy syndrome, wound complications, and hemorrhage (Chen and Cleck, 2009). At present, there is no systematic analysis of the toxicity of such a kind of combination. This review will focus on the severe treatment-related adverse events (trAEs) and irAEs of the concomitant use of ICIs and antiangiogenic agents.

## Material and Methods

### Search Strategy and Eligibility

The study was performed according to the “PRISMA” statement. Search was done on 31 May 2019. PubMed and Embase databases were searched for relevant literatures published in English using MeSH and keywords “nivolumab,” “pembrolizumab,” “atezolizumab,” “avelumab,” “ipilimumab,” “durvalumab,” “immune checkpoint inhibition” or “immune checkpoint inhibitors,” combined with “bevacizumab,” “ramucirumab,” “anlotinib,” “apatinib,” “axitinib,” “cabozantinib,” “cediranib,” “fruquintinib,” “lenvatinib,” “motesanib,” “nintedanib,” “pazopanib,” “regorafenib,” “sorafenib,” “sunitinib,” “vandetanib,” “aflibercept,” or “endostar.” Studies included in this review were limited to clinical trial of any phase, retrospective study, or case report involving adult patients with solid tumors. Only original articles were included. Duplicates, conference abstracts or poster presentations, commentaries, reviews, and secondary reporting of clinical trials were excluded.

Studies involving concurrent treatment of ICIs and antiangiogenic agents were eligible. The study should properly describe the safety of the combination treatment. Studies not describing toxicity or the timing of antiangiogenic therapy in relation to ICIs were excluded. AEs should be assessed according to the National Cancer Institute Common Terminology Criteria for Adverse Events (CTCAE). If not, authors rated them accordingly. When more than one article reported the same trial, the most recent data was used. When patients in case report were from the same cohort of a clinical trial and were reported with the same AEs, the case report was excluded. All relevant articles underwent evaluation for eligibility by two independent authors (LG and XY) and then were verified by senior author (HZ and CY). Titles and abstracts were preliminary screened. Subsequently, full-text reading was used to check whether the study met inclusion or exclusion criteria.

### Data Extraction

Two authors (LG and XY) collected all data for included studies. Data was sought on authors, year of publication, study type, number of patients, as well as the type, dose, and treatment duration of ICIs and antiangiogenic agents. Tumor types and stages, follow-up time, toxicity, and management were also collected. Only grade 3–5 trAEs and irAEs were included for analysis.

## Results

### Included Studies and Overview

**Figure 1:**
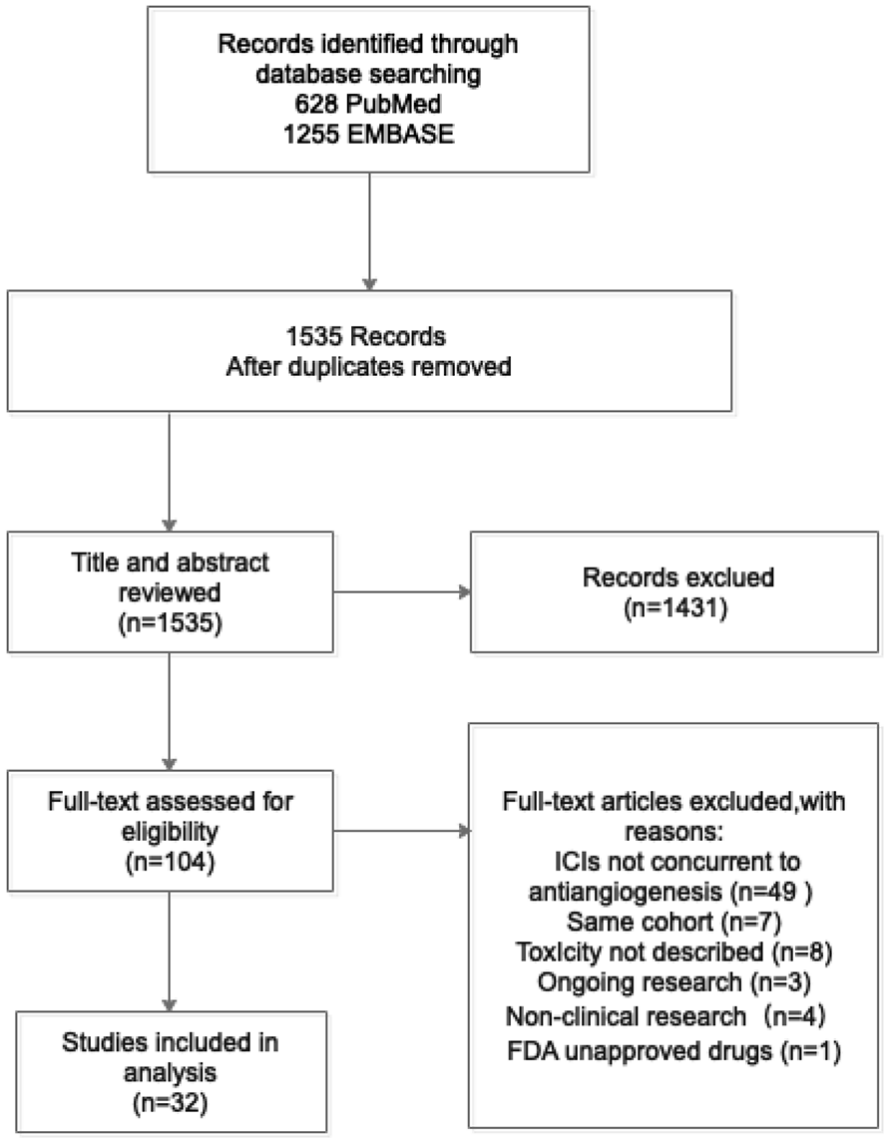
Search flow diagram.

We initially identified a total of 1,883 references from database search. There were 348 papers excluded due to duplication, and the remaining 1,535 references were read with title and abstract. Subsequently, 104 relevant articles were further assessed for eligibility by full-text reviewing. Finally, 32 articles meeting the inclusion criteria were included into this systematic review (**Figure 1**). Among them, there were 17 prospective studies (n = 2186), 5 retrospective studies (n = 104), and 10 case reports (n = 34), with the median number of patient as 70 per study. Studies of the combination of ICIs and anlotinib, fruquintinib, motesanib, nintedanib, regorafenib, vandetani, or aflibercept were not found. The concurrent use of ICIs and mAbs was reported in 15 studies (**Table 1**), while concurrent use of ICIs and TKIs was in 17 studies (**Table 2**).

**Table 1:** Included articles with concurrent ICIs and antiangiogenic mAbs.

Authors	Study year	Study type	Patients (n)	Compounds and dosage	Treatment timing	Primary tumor	Follow-up (median time)	Toxicity (≥3)
Wallin et al.	2016	Prospective	10	Bevacizumab 15 mg/kg i.v./3 w * 1 cycle, and then atezolizumab 20 mg/kg i.v., bevacizumab 15 mg/kg i.v./3 w	Renal cell carcinoma (RCC)	Advanced; metastatic	17.2 months	Y
McDermott et al.	2018	Phase 2 trial	101	Atezolizumab 1,200 mg i.v., bevacizumab 15 mg/kg i.v./3 w	RCC	Advanced; metastatic	20.7 months	Y
Rini et al.	2019	Phase 3 trial	451	Atezolizumab 1,200 mg i.v., bevacizumab 15 mg/kg i.v./3 w	RCC	Advanced; metastatic	15 months	Y
Reck et al.	2019	Phase 3 trial	394	Atezolizumab + bevacizumab + carboplatin + paclitaxel (atezolizumab 1,200 mg i.v., bevacizumab 15 mg/kg i.v./3 w)	Non-squamous non-small-cell lung cancer (NSCLC)	Chemotherapy-naïve metastatic	19.6 months	Y
Wu et al.	2017	Case report	1	Pembrolizumab + bevacizumab + cisplatin + gemcitabine (pembrolizumab 1 mg/kg i.v., bevacizumab 4 mg/kg i.v.)	Urothelial carcinoma (UC)	Recurrent	NR	N
Gadgeel et al.	2018	Phase 1 trial	24	Pembrolizumab + bevacizumab + carboplatin + paclitaxel (pembrolizumab 2(n = 11) or 10 mg/kg (n = 13) i.v., bevacizumab 15 mg/kg i.v./3 w * 4 cycles, and then pembrolizumab + bevacizumab for 2 years)	Non-squamous NSCLC	Stage IIIB/IV without EGFR mutations or ALK translocations	16.4 months	Y
Blumenthal et al.	2016	Retrospective	10	Pembrolizumab 150 mg i.v., bevacizumab, dosage NR i.v.,/3 w	Central nervous system (CNS) tumor	Recurrent	NR	N
Kurz et al.	2018	Retrospective	28	Pembrolizumab 2 mg/kg i.v./3 w (n = 19), or nivolumab 3 mg/kg i.v./2 w (n = 12), bevacizumab 10 mg/kg i.v./2 w (n = 28)	High-grade gliomas (HGGs)	Recurrent	NR	N
Mantica et al.	2018	Retrospective	43	Nivolumab 3 mg/kg i.v./2 w, bevacizumab, dosage NR	HGGs	Advanced	6.4 months	Y
Kanda et al.	2016	Phase 1b trial	6	Nivolumab+paclitaxel+carboplatin+bevacizumab (nivolumab 10 mg/kg i.v., bevacizumab 15 mg/kg i.v./3 w * 6 cycles, and then pembrolizumab + bevacizumab maintain)	Non-squamous NSCLC	Stage IIIB without indication for definitive thoracic radiotherapy; stage IV; recurrent	7.54 months	Y
Normann et al.	2019	Prospective	5	Nivolumab 3 mg/kg i.v./2 w, bevacizumab dosage NR	Platinum resistant ovarian cancer	Recurrent	30 weeks	Y
Shirali et al.	2016	Case report	1	Nivolumab 3 mg/kg i.v., bevacizumab 15 mg/kg i.v./3 w)	NSCLC	Progression	NR	Y
Hodi et al.	2014	Prospective	46	Ipilimumab 10 mg/kg i.v./3 w *4 cycles, and then 10 mg/kg i.v./12 w + bevacizumab 7.5 mg/kg (cohort 1) or 15 mg/kg (cohort 2) i.v./3 w; ipilimumab 3 mg/kg i.v./3 w *4 cycles, and then 3 mg/kg i.v./12 w + bevacizumab 7.5 mg/kg (cohort 3) or 15 mg/kg (cohort 4) i.v./3 w	Melanoma	Unresectable stage III; stage IV	17.3 months	Y
Carter et al.	2016	Case series	20	Ipilimumab 3 mg/kg i.v./3 w *4 cycles, and then 3 mg/kg i.v./12 w, bevacizumab 10 mg/kg i.v./2 w	Glioblastoma	Grade IV disease or recurrent astrocytoma (grade II); progression or after first-line therapy	≥12 weeks	Y
Arkenau et al.	2018	Phase 1 trial	26	Pembrolizumab 200 mg i.v. d1, ramucirumab 8 mg/kg i.v. d1, d8/3 w	Biliary tract cancer (BTC)	Advanced; metastatic	15.7 months	Y

**Table 2:** Included articles with concurrent ICIs and TKIs.

Authors	Study year	Study type	Patients (n)	Compounds and dosage	Primary tumor	Treatment timing	Follow-up (median time)	Toxicity (≥3)
Atkins et al.	2018	Phase 1b trial	52	Axitinib 3,5 or 7 mg p.o. bid continuously, (median dose: 8.8 mg/day), pembrolizumab 2 mg/kg i.v. d8/3 w	RCC	Advanced	20.4 months	Y
Rini et al.	2019	Phase 3 trial	429	Axitinib 5 mg (2–10 mg) p.o. bid continuously, pembrolizumab 200 mg i.v./3 w	RCC	Advanced, recurrent	12.8 months	Y
Wilky et al.	2019	Phase 2 trial	33	Axitinib 5 mg (2–10 mg) p.o. bid continuously, pembrolizumab 200 mg i.v. d8/3 w up to 2y	Sarcomas, including alveolar soft-part sarcoma (ASPS)	Advanced; metastatic	14.7 months	Y
Choueiri et al.	2018	Phase 1b trial	55	Axitinib 5 mg p.o. bid, d1–7 (lead-in period), axitinib 5 mg p.o. bid continuously, avelumab 10 mg/kg i.v./2 w	RCC	Advanced	52.1 weeks	Y
Motzer et al.	2019	Phase 3 trial	434	Axitinib 5 mg p.o. bid, avelumab 10 mg/kg i.v./2 w	RCC	Advanced	11.6 months	Y
Qiao et al.	2018	Case report	1	Pazopanib + pembrolizumab + RAK cells (pazopanib 200 mg p.o. qd for 2 days, 400 mg qd for 5 days, then 600 mg qd up to now, pembrolizumab 100 mg i.v./3 w)	Primary hepatic angiosarcoma (PHA)	Advanced	About 15 months	N
Amin et al.	2018	Phase 1 trial	20(P+N)33(S+N)	Pazopanib 800 mg p.o. qd, nivolumab 2 mg/kg i.v./3 w; sunitinib 50 mg p.o. qd/4 weeks on and 2 weeks off, nivolumab 2 mg/kg i.v./3 w	RCC	Advanced	27.1 months (P+N); 50 months (S+N)	Y
Paoluzzi et al.	2016	Retrospective	18	Pazopanib 400–800 mg p.o. qd, nivolumab 3 mg/kg i.v./2 w	Sarcomas	Relapsed metastatic; unresectable	≥13 months	Y
Yu-Li Su et al.	2017	Case report	1	Pazopanib 400 mg p.o. qd continuingly, nivolumab 3 mg/kg i.v./2 w	RCC	Metastatic	≥4 months	N
Chen et al.	2017	Case report	1	Sorafenib 200 mg p.o. bid, pembrolizumab 2 mg/kg i.v. d1/3 w (4 w starting in cycle 3)	Hepatocellular carcinoma (HCC)	End-stage	NR	N
Feng et al.	2017	Case series	6	Sorafenib 200 mg p.o. bid, nivolumab 3 mg/kg i.v. d1/3 w	HCC	Advanced	NR	N
Mahmoud et al.	2016	Case report	1	Sunitinib 50 mg p.o. qd/4 weeks on and 2 weeks off, nivolumab NR	RCC	Metastatic	≥11 months	N
Lee et al.	2017	Phase 1 trial	14	Cediranib 20/30 mg p.o. qd, + durvalumab 10 mg/kg i.v./2 w; cediranib 20 mg p.o. qd/5 days on and 2 days off, + durvalumab 1,500 mg i.v./4 w	Solid tumors	Recurrent; metastatic	NR	Y
Zhao et al.	2019	Case report	1	Apatinib 500 mg p.o. qd, nivolumab 3 mg/kg i.v./2 w	Liver carcinosarcoma	Advanced	About 15 months	Y
Makker et al.	2019	Phase 2 trial	53	Lenvatinib 20 mg p.o. bid, pembrolizumab 200 mg i.v./3 w	Endometrial cancer	Metastatic	13.3 months	Y
Iyer et al.	2018	Retrospective	12	Lenvatinib 20 mg p.o. bid, pembrolizumab 200 mg i.v./3 w	Anaplastic thyroid carcinoma (ATC)	Progression	13.74 months (8.14 + 5.6)	Y
Bhat et al.	2019	Case report	1	Cabozantinib, nivolumab, dosage NR	RCC	Metastatic	NR	N

**Figure 2:**
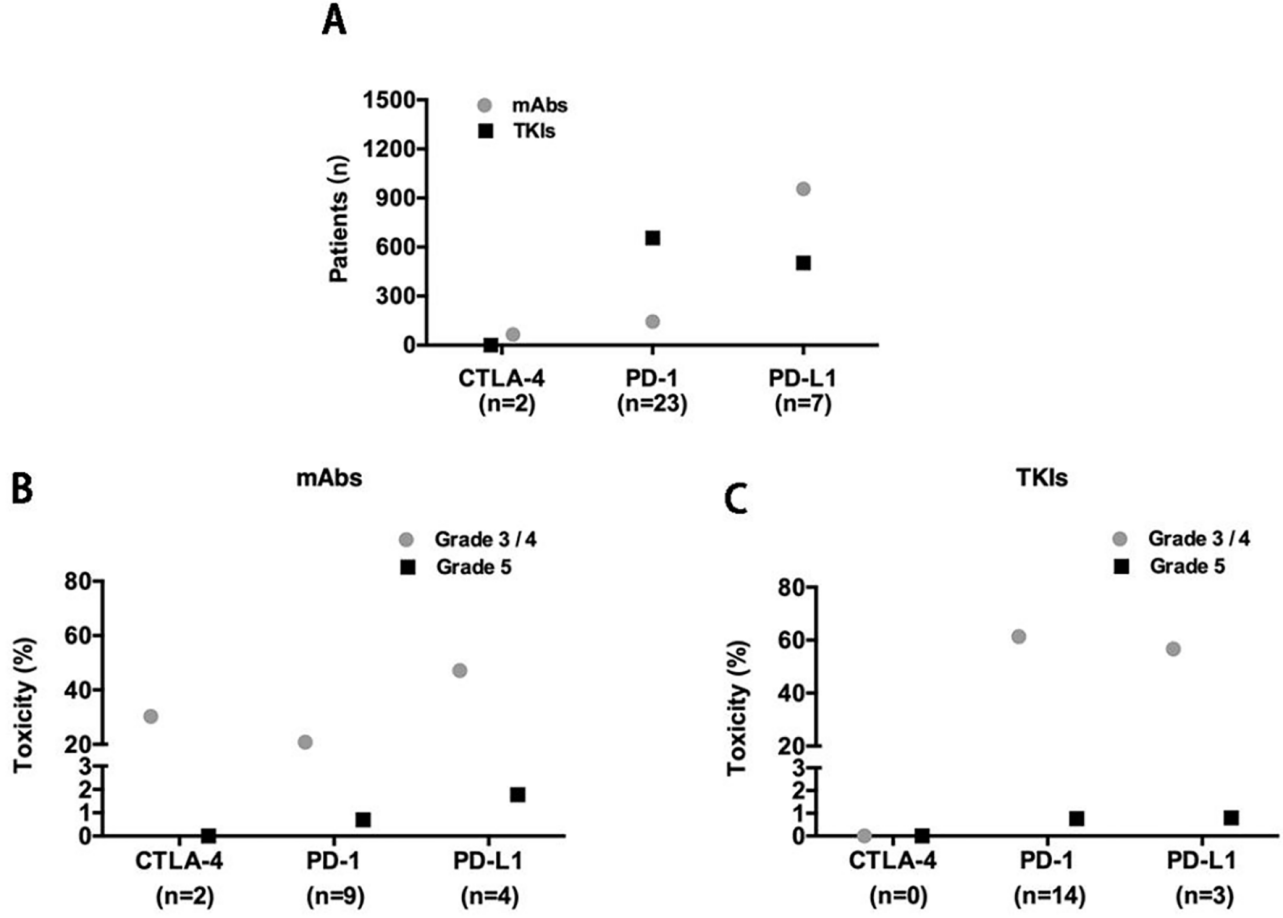
**(A)** Included studies and patients; X-axis: n, number of included studies. Severe trAE evaluation of concurrent use of antiangiogenic mAbs **(B)** or TKIs **(C)** with each class of ICIs.

The reported treatment-related toxicities of included studies were listed in **Table 3**. When ICIs combined with mAbs (n = 1166), severe toxicity reported as grade 3/4 and grade 5 AEs was observed in 501 (43%) and 18 (1.5%) patients, respectively (**Figures 2A, B**), while for ICIs plus TKIs (n = 1158), grade 3/4 and grade 5 AEs were in 687 (59.3%) and 9 (0.8%) patients, respectively (**Figures 2A, C**). Overall, the total incidence of severe trAEs associated with ICIs plus mAbs was lower than that of ICIs plus TKIs (**Figures 2B, C**).

**Table 3:** Treatment-related toxicity as observed within the included articles.

Antiangiogenic agents	ICIs	Study	Median treatment duration	Patients (n)	Grade 3 (n) trAE/irAE	Grade 4 (n) trAE/irAE	Grade 5 (n) trAE/irAE	Total toxicity (≥3)	Management
Bevacizumab				1,140	494		18	512	
	Ipilimumab	Hodi et al.	NR	46	ALT (n = 2), AST (n = 2), abdomen pain (n = 2), adrenal insufficiency (n = 2), allergic reaction (n = 1), colitis (n = 2), endocrine-other (n = 1), fatigue (n = 1), head or headache (n = 1), hemorrhage-other (n = 1), hepatic-other (n = 1), hypertension (n = 4), hyponatremia (n = 2), lipase (n = 2), lymphopenia (n = 1), mucostomatitis by exam, oral cavity (n = 1), rash or desquamation (n = 2), thrombosis or thrombus or embolism (n = 1), vascular-other (n = 1). Among them, 5 trAEs were observed in cohort 1, 8 were in cohort 2, 6 were in cohort 3, and 10 in cohort 4.	Hepatic-other (n = 1), proteinuria (n = 2) All above were observed in cohort 2 Number of patient was 13 (grade 3/4)	0	13	NR
		Carter et al.	65% patients complete four cycles	20	Diarrhea (n = 1), abscess formation (dental, uterine, diverticular) (n = 3), intracerebral bleed (n = 1), pulmonary embolism (n = 2)	0	0	7	Three abscess were managed surgically; corticosteroids (diarrhea), dosage NR; NO discontinued treatment.
	Pembrolizumab	Gadgeel et al.	Pemb: 10 doses (30 weeks)	24	Thrombocytopenia (n = 1), neutrophil count decreased (n = 1), white blood cell count decreased (n = 2)/colitis (n = 1), pneumonitis (n = 1), pancreatitis (n = 1). Grade 3 trAEs occurred in 10 (42%) and 10 (40%) patients with or without bevacizumab, respectively. Grade 3 irAEs and infusion reactions occurred in 5 (20.8%) and 1 (4%) patients with or without bevacizumab, respectively.	0	0	10	Discontinuation: pembrolizumab 2 mg/kg group (n = 2, 18%); 10 mg/kg group (n = 3, 23%)
		Blumenthal et al.	Pemb: 3 doses (9 weeks)	10	NR	NR	NR	0	Steroids weaned off or minimal 2 mg/d
		Wu et al.	11 cycle (about 7.7 months)	1	NR	NR	NR	0	A mild immune-related skin was resolved completely with anti-histamines.
	Pembrolizumab or nivolumab	Kurz et al.	NR	28	0	0	0	0	On steroids when pembrolizumab initiated: n = 17 (55%), dosage NR Discontinuation: n = 1 (3%)
	Nivolumab	Mantica et al.	8 cycle (about 16 weeks)	43	Pneumonitis (n = 1) / irAEs (including colitis and pneumonitis): n = 3	Pneumonitis (n = 2), colitis (n = 1)	0	4	Discontinuation: n = 4 (8%)
		
		Kanda et al.	NR	6	White blood cell count decreased (n = 3), neutrophil count decreased (n = 6), lymphocyte count decreased (n = 1), anemia (n = 1), platelet count decreased (n = 2), febrile neutropenia (n = 1)/select adverse events (those with a potential immunologic cause) (n = 0); number of patient was 6.		0	6	No discontinuation. NR
		Normann et al.	Bev: 16 weeks Nivo: 12 weeks	5	Hepatitis (n = 1) There was a tendency toward increased toxicity when using concomitant bevacizumab [2 (40%) of 5 vs. 1 (11%) of 9].	0	Intestinal perforation (n = 1); Believed to cause by bevacizumab	3	Grade 2 events continued treatment after administration of steroids (dosage NR) Discontinuation because of nivolumab: n = 2 (14%)
		Shirali, et al.	10 months	1	Acute interstitial nephritis (n = 1)	NR	NR	1	Hospitalization: methylprednisolone 125 mg i.v. for 3 days, followed by prednisone 60 mg/d p.o., which was tapered over the next month.
	Atezolizumab	Wallin et al.	Atez: 15.9 months	10	Hypertension (n = 3), acute respiratory failure (n = 1), hypercalcemia (n = 1), abdominal pain (n = 1)/n = 0		0	6	NR
		McDermott et al.	Bev: 10.3 months Atezb: 11.8 months	101	Fatigue (n = 2), diarrhea (n = 4), nausea (n = 1), palmar–plantar erythrodysaesthesia syndrome (PPE) (n = 2), decreased appetite (n = 2), stomatitis (n = 2), headache (n = 1), arthralgia (n = 1), proteinuria (n = 8)/elevated liver enzymes or hepatitis (n = 4). TrAEs significantly increased with addition of bevacizumab (40 vs. 17%), but frequencies of irAEs were similar (5 [5%] of 101 vs. 3[3%] of 103).		Intracrinal hemorrhage (n = 1)	41	Discontinuation: n = 9 (9%) Dose modification or interruption: n = 61 (60%)
		Rini et al.	12 months	451	Hypertension (n = 63), fatigue (n = 6), hypothyroidism (n = 1), diarrhea (n = 7), proteinuria (n = 15), rush (n = 3), arthralgia (n = 10), decreased appetite (n = 2), nausea (n = 1), stomatitis (n = 2), mucosal inflammation (n = 1), anemia (n = 1), thrombocytopenia (n = 3), neutropenia (n = 2)/rush (n = 3), hypothyroidism (n = 1), hyperthyroidism (n = 1), LFT abnormalities (n = 13), colitis (n = 4), pneumonitis (n = 4). Frequency of trAEs was lower than that of sunitinib [182 (40%) of 451 vs. 240 (54%) of 446].		Cerebral infarction (n = 1, with known hypercholesterolaemia), intracranial hemorrhage (n = 1, following a fall), adrenal insufficiency (n = 1, with a history of coronary artery disease and myocardial infarction), multiple organ dysfunction syndrome (n = 1, following a post-radiation ulcer with cecum perforation), sepsis (n = 1, following pneumonia)	187	Discontinuation:treatment regimen n = 24 (5%), any treatment component n = 53 (12%) Systemic corticosteroids: n = 74 (16%) High-dose systemic corticosteroids (prednisone ≥40 mg/d or equivalent): n = 42 (9%)
		Reck et al.	Bev: 6.7 months Atez: 8.2 months	394	Peripheral neuropathy (n = 11), nausea (n = 15), fatigue (n = 13), anemia (n = 24), decreased appetite (n = 10), diarrhea (n = 11), neutropenia (n = 54), hypertension (n = 25), arthralgia (n = 3), asthenia (n = 5), epistaxis (n = 4), vomiting (n = 6), decreased platelet count (n = 20), myalgia (n = 2), thrombocytopenia (n = 16), proteinuria (n = 10), decreased neutrophil count (n = 34), rush (n = 5), stomatitis (n = 4), febrile neutropenia (n = 33), decreased white blood cell count (n = 13), decreased weight (n = 4), alt increased (n = 4), dehydration (n = 8), AST increased (n = 4), leukopenia (n = 7), hypokalemia (n = 7), pulmonary embolism (n = 7), hyponatremia (n = 8), pneumonia (n = 7), pneumonitis (n = 4), colitis (n = 5), transaminases increased (n = 4), cerebrovascular accident (n = 1), sepsis (n = 1)/rash (n = 9), hepatitis (laboratory abnormalities) (n = 16), hypothyroidism (n = 1), hyperthyroidism (n = 1), pneumonitis (n = 6), colitis (n = 5), hepatitis (diagnosis) (n = 4), adrenal insufficiency (n = 1), pancreatitis (n = 2), hypophysitis (n = 1), nephritis (n = 1), ocular inflammatory toxicity (n = 1), myositis (n = 1), encephalitis (n = 1), meningoencephalitis (n = 1); information was from an article reporting the same trial (Socinski et al., 2018). TrAEs elevated with addition of bevacizumab or atezolizumab (56.7 vs. 43%, 56.7 vs. 48.5%). But the addition of bevacizumab did not significantly increased irAEs (12.5 vs. 9.5%).		Febrile neutropenia (n = 3), hemoptysis (n = 3), pulmonary hemorrhage (n = 2), cerebrovascular accident (n = 1), aortic dissection (n = 1), intestinal obstruction (n = 1). Information was from an article reporting the same trial (Socinski et al., 2018). Treatment-related death elevated with addition of bevacizumab (2.8 vs. 1%), but the addition of atezolizumab did not significantly increased it (2.8 vs. 2.3%).	234	Discontinuation or interruption No dose reduction for atezolizumab or bevacizumab Steroids, dosage NR
Ramucirumab				26	7		0	7	
	Pembrolizumab	Arkenau et al.	Ramu: 9 weeks Pemb: 9.3 weeks	26	Hypertension (n = 5), alanine aminotransferase increased (n = 1), aspartate aminotransferase increased (n = 1)	0	0	7	Discontinuation: n = 1 (3.8%)
Apatinib				1	1		0	1	
	Nivolumab	Zhao et al.	About 7 months	1	Elevated aminotransferases (n = 1)	NR	NR	1	Discontinued and received liver-protecting drugs with magnesium isoglycyrrhizinate injection and transmetil for 3 weeks.
Axitinib				1003	594		8	602	
	Pembrolizumab	Atkins et al.	14.5 months	52	Fatigue (n = 5), diarrhea (n = 5), hypertension (n = 12), increased alanine aminotransferase concentration (n = 4), decreased appetite (n = 1), nausea (n = 1), palmar–plantar erythrodysaesthesia (n = 2), increased aspartate aminotransferase concentration (n = 2), weight decreased (n = 2), proteinuria (n = 1), oral pain (n = 1), headache (n = 2), vomiting (n = 1), dizziness (n = 1)/diarrhea (n = 4), increased alanine aminotransferase concentration (n = 2), increased aspartate aminotransferase concentration (n = 2), fatigue (n = 2), weight decreased (n = 1), colitis (n = 1), lymphocyte count decreased (n = 1)	Hyperuricemia (n = 1)/hyperuricemia (n = 1)	0	34	Axitinib dose modification + symptomatic treatment: axitinib starting dose: 5 mg bid; dose level-1: 3 mg bid; dose level-2: 2 mg bid; permanently discontinued. For pembrolizumab: hold treatment until toxicity was <grade 2; discontinue if toxicity does not resolves within 12 weeks of last dose or inability to reduce corticosteroids to 10 mg or less of prednisone or equivalent per day within 12 weeks; permanently discontinue
		Rini et al.	Pemb+axi: 8.3 months Pemb: 9.2 months Axi: 9.6 months	429	Diarrhea (n = 31), hypertension (n = 91), hypothyroidism (n = 1), fatigue (n = 10), palmar–plantar erythrodysesthesia (n = 22), alanine aminotransferase increased (n = 52), dysphonia (n = 1). Aspartate aminotransferase increased (n = 29), decreased appetite (n = 9), nausea (n = 2), proteinuria (n = 11), stomatitis (n = 3), mucosal inflammation (n = 4), pruritus (n = 1), arthralgia (n = 3), hyperthyroidism (n = 4), asthenia (n = 6), rash (n = 1), dysgeusia (n = 1), vomiting (n = 1), platelet count decrease (n = 1), anemia (n = 1), neutrophil (n = 1), neutrophil count decreased (n = 1)/hypothyroidism (n = 1), hyperthyroidism (n = 5), adrenal insufficiency (n = 3), hepatitis (n = 10, pneumonitis (n = 2), thyroiditis (n = 1), colitis (n = 8), severe skin reactions (n = 5), infusion reactions (n = 1), nephritis (n = 1), hypophysitis (n = 4), myasthenic syndrome (n = 2), myositis (n = 1), myocarditis (n = 2), pancreatitis (n = 2), type 1 diabetes mellitus (n = 1)		Myasthenia gravis (n = 1), myocarditis (n = 1), necrotizing fasciitis (n = 1), pneumonitis (n = 1)/myasthenia gravis (n = 1), myocarditis (n = 1), pneumonitis (n = 1) Incidence of treatment-related death was lower than that of sunitinib [4(0.9%) vs. 7 (1.6%)].	270	Interruption: n = 267 (62.2%) Discontinuation of both pembrolizumab and axitinib: n = 35 (8.2%) Dose reduction of axitinib: n = 86 (20%) Steroids, dosage NR
		Wilky et al.	NR	33	Oral mucositis (n = 1), nausea or vomiting (n = 2), diarrhea (n = 1), abdominal pain or dyspepsia (n = 1), hypertension (n = 5), hemoptysis (n = 1), pneumothorax (n = 1), seizures (n = 2)/hyperglycemia (n = 1), autoimmune hepatitis (n = 1), autoimmune colitis (n = 1), autoimmune arthritis (n = 2)	Elevated ALT, AST, or AP (n = 1), hypertriglyceridemia or hyperlipidemia (n = 1)	0	16	Axitinib dose modification + symptomatic treatment: axitinib starting dose: 5 mg bid. If grade 2 or greater toxicity, dose level-1: 4 mg bid; dose level-2: 3 mg bid; dose level-3: 2 mg bid; permanently discontinued. Steroids and discontinuation of study treatment: n = 3 (9%). One patient with autoimmune arthritis was also given methotrexate and hydroxychloroquine.
	Avelumab	Choueiri et al.	Axi: 66.6 weeks Ave: 66.0 weeks	55	Diarrhea (n = 2), hypertension (n = 16), fatigue (n = 2), PPE syndrome (n = 4), ALT increased (n = 4), rush (n = 1), AST increased (n = 1), amylase increased (n = 3), decreased appetite (n = 1), mucosal inflammation (n = 1), infusion-related reaction (n = 1), lipase increased (n = 1), nausea (n = 1), arthralgia (n = 1), weight decreased (n = 1), proteinuria (n = 2), hypophosphatemia (n = 2), blood triglycerides increased (n = 1), dehydration (n = 1), pain in extremity (n = 1), drug eruption (n = 1), dyslipidemia (n = 1), urticaria (n = 1), venous thrombosis (n = 1)/rash (n = 2), hepatitis (n = 2), colitis (n = 1)	Amylase increased (n = 1), lipase increased (n = 3), hematoma (n = 1), pulmonary embolism (n = 1)	Myocarditis (n = 1)	33	Dose interruption of avelumab: n = 1 (1.8%) Discontinuation of avelumab: n = 7 (13%) Discontinuation of axitinib: n = 4 (7%) Dose reductions of axitinib: n = 28 (51%) Steroids, dosage NR
		Motzer et al.	Axi: 9.0 months Ave: 8.6 months	434	Diarrhea (n = 22), hypertension (n = 106), fatigue (n = 13), PPE syndrome (n = 25), dysphonia (n = 2), nausea (n = 3), hypothyroidism (n = 1), stomatitis (n = 8), decreased appetite (n = 7), chills (n = 1), mucosal inflammation (n = 5), alanine aminotransferase increased (n = 21), rash (n = 2), dyspnea (n = 6), arthralgia (n = 1), infusion-related reaction (n = 7), aspartate aminotransferase increased (n = 12), weight decreased (n = 7), vomiting (n = 1), asthenia (n = 5), thrombocytopenia (n = 1), anemia (n = 1), neutropenia (n = 1)/n = 39, events NR		Sudden death (n = 1), myocarditis (n = 1), necrotizing pancreatitis (n = 1)/n = 0	249	Discontinuation of both avelumab and axitinib: n = 33 (7.6%) Dose reduction of axitinib: n = 183 (42.2%) High-dose glucocorticoids (≥40 mg total daily dose of prednisone or equivalent): n = 48 (11.1%)
Cabozantinib				1	0		0	0	
	Nivolumab	Bhat et al.	NR	1	NR	NR	0	0	NR
Cediranib				14	7		0	7	
	Durvalumab	Lee et al.	>15 months	14	(1) Once-daily cediranib: lymphopenia (n = 1), anemia (n = 2), nausea (n = 1), diarrhea (n = 3), colitis (n = 1), fatigue (n = 1), headache (n = 1), hypertension (n = 3), pulmonary thromboembolism (n = 1), pulmonary hypertension (n = 1). Number of patient was 7; (2) intermittent cediranib:fatigue (n = 1)	(1) Once-daily cediranib: lymphopenia (n = 1), pulmonary thromboembolism (n = 1); (2) intermittent cediranib: hypertension (n = 1)	NR	7	Discontinued or dose reduced of daily cediranib: n = 7 (87.5%) Systemic corticosteroids, dosage NR
Lenvatinib				58	40		1	41	
	Pembrolizumab	Makker et al.	NR	53	Fatigue (n = 3), diarrhea (n = 4), palmar–plantar erythrodysesthesia syndrome (n = 3), hypertension (n = 18), proteinuria (n = 1), oral pain (n = 1), dehydration (n = 1), increased aspartate aminotransferase (n = 1), anemia (n = 1), hyponatremia (n = 2), increased lipase (n = 1), increased alanine aminotransferase (n = 1), prolonged electrocardiogram qt interval (n = 1), hypocalcaemia (n = 1), acute kidney injury (n = 2), pulmonary embolism (n = 2), syncope (n = 2), adrenal insufficiency (n = 1), cardiac failure (n = 1), colitis (n = 1), dysarthria (n = 1), hypertensive encephalopathy (n = 1), ischemic colitis (n = 1), neutropenia, pancreatitis (n = 1), retinal vein occlusion (n = 1), small intestinal obstruction (n = 1), upper abdominal pain (n = 1)/n = 30, irAEs (including skin, endocrine, gastrointestinal, pulmonary, hepatic, and renal adverse events), but grade NR	0	Intracranial hemorrhage (n = 1)	37	Discontinued: n = 5 (9%) High-dose glucocorticoids (≥40 mg/d of prednisone or equivalent): n = 3 (10%)
		Iyer et al.	5.6 months	5	Fatigue (n = 1), hypokalemia (n = 1), weakness (n = 1), altered mental status (n = 1), hypophosphatemia (n = 1)/2 patients had mild irAEs, including a grade 2 hepatic	0	0	4	Grade 2 colitis: n = 1(20%), budesonide, dosage NR, and continued pembrolizumab Grade 2 hepatitis: n = 1(20%), high dose of prednisone, and discontinued pembrolizumab.
Pazopanib				40	18		0	18	
	Pembrolizumab	Qiao et al.	About 15 months	1	NR	NR	NR	0	NR
	Nivolumab	Amin et al.	Pazo: 13.9 months Nivo: 15.1 months	20	Fatigue (n = 3), diarrhea (n = 4), hypertension (n = 2), increased alt (n = 4), increased AST (n = 4), hypothyroidism (n = 1), arthralgia (n = 1)/endocrine (n = 2), gastrointestinal (n = 4), hepatic (n = 4)		0	14	Discontinuation: n = 5 (25%) Systemic corticosteroid: n = 12(60%), including prednisone [n = 11 (55%)], dexamethasone [n = 2 (10%)], and methylprednisolone [n = 2 (10%)].
		Paoluzzi et al.	Nivo: 8 cycles (16 weeks)	18	AST elevation (n = 1), ALT elevation (n = 3), alkaline bilirubin elevation (n = 2), pneumonitis (n = 1), colitis (n = 1). The total number of patient who suffered grade 3/4 trAEs was 4. The frequencies of trAEs significantly increased with addition of pazopanib [4 (22%) of 18 vs. 0 (0%) of 10].	Bilirubin elevation (n = 1), AST elevation (n = 1)	0	4	Discontinuation of both nivolumab and pazopanib: n = 4 (22%), among which, two patients restarted on treatment with both drugs, while one patient restart pazopanib only High-dose steroids (prednisone 1 mg/kg/daily), with a slow taper over about 2 months: n = 3 One patients needed intubation.
		Yu-Li Su et al.	4 months	1	NR	NR	NR	0	NR
Sorafenib				7	0		0	0	
	Pembrolizumab	Chen et al.	NR	1	NR	NR	NR	0	To avoid tumor rupture, the schedule of pembrolizumab was changed to every 4 weeks starting in cycle three.
	Nivolumab	Feng et al.	Nivo: 7.1 cycles	6	NR	NR	NR	0	NR
Sunitinib				34	27		0	27	
	Nivolumab	Amin et al.	Suni: 28 months Nivo: 45.1 months	33	Fatigue (n = 3), diarrhea (n = 3), nausea (n = 1), hypertension (n = 6), decreased appetite (n = 1), increased alt (n = 6), increased AST (n = 3), blood creatinine increased (n = 2), vomiting (n = 1)/skin (n = 2), gastrointestinal (n = 3), hepatic (n = 8), renal (n = 4), pulmonary (n = 1)		0	27	Discontinuation of both nivolumab and sunitinib: n = 13(39.4%) Systemic corticosteroid n = 13 (39.4%) (prednisone, dexamethasone, and methylprednisolone)
		Mahmoud et al.	Suni: ≥11 months Nivo: ≥8 months	1	NR	NR	0	0	NR

### Toxicity of Concurrent ICIs and Antiangiogenic mAbs (Bevacizumab and Ramucirumab)

#### Anti-CTLA-4 (Ipilimumab)

One prospective study (Hodi et al., 2014) and one case series (Carter et al., 2016) were identified (**Table 1**), examining concurrent ipilimumab and bevacizumab in melanoma and glioblastoma, respectively. The median dose of bevacizumab ranged from 7.5 to 15 mg/kg, and the dose of ipilimumab was 3 or 10 mg/kg every 3 weeks. Hodi et al. reported a total of 32 grade 3/4 trAEs in 13 patients, including one grade 4 hepatic and two grade 4 proteinuria. Among them, grade 3/4 trAEs in four cohorts were 5 (15.6%), 11 (34.4%), 6 (18.8%), and 10 (31.3%), respectively (**Table 3**). It seemed that the incidence of severe trAEs tended to elevate with the increase dose of bevacizumab. But it did not seem to increase the incidence of dermatologic or gastrointestinal side effects such as colitis, which were more concerning for ipilimumab treatment (Hodi et al., 2014). In addition, one case series reporting the combination regimen in glioblastoma observed seven grade 3 trAEs. However, all immune-related toxicities were manageable with corticosteroids, without diagnosing of endocrinopathies (Carter et al., 2016).

In summary, limited available data indicated potential toxicity of concurrent ipilimumab and bevacizumab. But at least, it did not seem to increase the incidence of some special interest irAEs. Data on the combination of ipilimumab and ramucirumab is lacking.

#### Anti-PD-1 (Pembrolizumab and Nivolumab)

For pembrolizumab, there were two phase 1 trials (Arkenau et al., 2018; Gadgeel et al., 2018), two retrospective studies (Blumenthal et al., 2016; Kurz et al., 2018), and one case report (Wu et al., 2017). Among them, four were concerning combined with bevacizumab, and one was with ramolumab. In the study of Gadgeel et al. where 24 patients with advanced non-squamous NSCLC received concurrent pembrolizumab, bevacizumab, and chemotherapy, grade 3 trAEs occurred in 10 (42%) patients, which was similar to patients treated without bevacizumab [10 (40%)]. But the grade 3 irAEs (colitis, pneumonitis, and pancreatitis) and infusion reaction occurred in five (20.8%) and one (4%) patients treated with or without addition of bevacizumab (**Table 3**) (Gadgeel et al., 2018). In the two retrospective studies, concomitant use of pembrolizumab and bevacizumab in recurrent central nervous system (CNS) tumor was well tolerated, with no significant toxicity (Blumenthal et al., 2016; Kurz et al., 2018). The only one case report by Wu et al., 2017 observed no grade 3–5 trAEs in one patient with urothelial carcinoma (UC) receiving such a concurrent regimen (Wu et al., 2017). There was only one phase 1 trial, treating a total of 26 patients with concurrent pembrolizumab and ramucirumab for advanced or metastatic biliary tract cancer (BTC). A total of seven (27%) grade 3 trAEs of 26 patients were observed, with hypertension accounting for 71% (**Table 3**) (Arkenau et al., 2018).

As for nivolumab, two retrospective studies on recurrent high-grade gliomas (HGGs) (Kurz et al., 2018; Mantica et al., 2018), one phase 1b trial and one case report on non-squamous NSCLC (Kanda et al., 2016; Shirali et al., 2016) and one prospective trial on platinum resistant ovarian cancer (Normann et al., 2019) were identified (**Table 1**). In the two retrospective studies (n = 71 patients), a total of four (5.6%) patients experienced grade 3/4 trAEs, among which there were three cases of irAEs including colitis and pneumonitis (**Table 3**) (Kurz et al., 2018; Mantica et al., 2018). The phase 1b trial of concurrent nivolumab, bevacizumab, and chemotherapy for NSCLC patients observed a total of 14 grade 3/4 trAEs of 6 patients. However, all of them were hematological AEs, and no grade 3/4 irAEs were reported (Kanda et al., 2016). The prospective trial by Normann et al. observed a grade 3 hepatitis, and one death (grade 5) of intestinal perforation which was believed to be caused by bevacizumab in recurrent ovarian cancer patients. They also found that there was a tendency to increase toxicity when using concomitant nivolumab and bevacizumab [2 (40%) of 5 vs. 1 (11%) of 9] (**Table 3**) (Normann et al., 2019). Besides, Shirali, et al. reported one event of grade 3 acute interstitial nephritis in a progressive NSCLC patient treated with concurrent nivolumab and bevacizumab (Shirali et al., 2016).

In summary, although the available data was limited, it suggested that concurrent use of pembrolizumab/nivolumab and bevacizumab is relatively safe. The data on the combination of pembrolizumab/nivolumab and ramucirumab is insufficient for conclusions.

#### Anti-PD-L1 (Atezolizumab)

Bevacizumab was combined with atezolizumab in four prospective studies (Wallin et al., 2016; McDermott et al., 2018; Reck et al., 2019; Rini et al., 2019b). Three were associated with unresectable or metastatic RCC, and one was about chemotherapy-naïve metastatic non-squamous NSCLC (**Table 1**). The median follow-up ranged from 15 to 20.7 months. In total, 468 severe trAEs (≥grade 3), including 17 treatment-related deaths (grade 5), were reported of 956 patients (**Table 3**). But our search did not find studies on concurrent atezolizumab and ramucirumab.

In patients with RCC, grade 3/4 trAEs were observed in 228 (40.6%) of 562 patients, and also 6 (1.1%) grade 5 trAEs were reported, consisting of 2 intracranial hemorrhage, 1 cerebral infarction, 1 adrenal insufficiency, 1 multiple organ dysfunction syndrome, and 1 sepsis (**Table 3**). An early clinical trial reported six grade 3/4 trAEs, but none of them were deemed related to atezolizumab (Wallin et al., 2016). McDermott et al found that concurrent atezolizumab and bevacizumab led to a significantly increase of the incidence of grade 3–5 trAEs (40 *vs*. 17%), but the incidence of irAEs was similar (5 *vs*. 3%) (McDermott et al., 2018). The phase 3 trial of Rini et al. observed that patients given atezolizumab plus bevacizumab had lower frequency of grade 3/4 trAEs than that of sunitinib (40 *vs*. 54%) (**Table 3**) (Rini et al., 2019b). Regarding NSCLC, the only one phase 3 trial suggested that when adding atezolizumab to bevacizumab and chemotherapy, grade 3/4 trAEs and treatment-related death (grade 5) slightly elevated, while the increase degree was higher when adding bevacizumab to atezolizumab and chemotherapy. However, the addition of bevacizumab did not significantly increase the incidence of irAEs (12.5 *vs*. 9.5%) (**Table 3**) (Reck et al., 2019). Information about the AEs was from an article that reporting the same trial (Socinski et al., 2018).

In summary, concurrent atezolizumab and bevacizumab might increase trAEs, but not irAEs. In addition, no unexpected patterns of toxicity emerged in the combination therapy. Data about the combination of atezolizumab and ramucirumab is not available.

### Toxicity of Concurrent ICIs and Antiangiogenic TKIs (Apatinib, Axitinib, Cabozantinib, Cediranib, Lenvatinib, Pazopanib, Sorafenib, Sunitinib)

#### Anti-PD-1 (Pembrolizumab, Nivolumab)

The concomitant use of pembrolizumab and antiangiogenic TKIs was examined in seven studies (Chen et al., 2017; Atkins et al., 2018; Iyer et al., 2018; Qiao et al., 2018; Makker et al., 2019; Rini et al., 2019a; Wilky et al., 2019), which were highly diverse in research and tumor types (**Table 2**). In a phase 1b study, where a total of 52 RCC patients received concurrent pembrolizumab and axitinib, grade 3/4 trAEs were observed in 34 patients. The most common trAEs, such as diarrhea (8%) and elevations in liver-enzyme levels (8%), seemed to be largely related to axitinib rather than a true irAE predominantly due to pembrolizumab (Atkins et al., 2018). Similarly, the phase 3 trial by Rini et al. reported 270 (51%) grade 3 or higher toxicities of 429 patients (**Table 3**), which were as expected on the basis of the known profiles of each drug. Although there were four (0.9%) patients died from trAEs, none of them related to hepatic adverse that might be more challenging due to the overlapping toxicities of axitinib and pembrolizumab. Moreover, combined group had fewer treatment-related death than sunitinib [4 (0.9%) *vs*. 7 (1.6%)] (Rini et al., 2019a). We also found a phase 2 trial of this combined regimen for soft-part sarcoma. A total of 16 grade 3/4 trAEs occurred in 33 patients, and grade 3/4 irAEs in 5 (15%) patients (Wilky et al., 2019). Two studies examining concurrent pembrolizumab and lenvatinib were identified. In a phase 2 trial of metastatic endometrial cancer, Makker et al. observed 36 (68%) patients with grade 3 trAEs and a grade 5 intracranial hemorrhage. Among them, there were 30 irAEs in total, but the grade was not described in detail (**Table 3**) (Makker et al., 2019). One retrospective study for progressive anaplastic thyroid carcinoma (ATC) reported four grade 3 trAEs of five patients and some mild irAEs (such as grade 2 hepatitis) (Iyer et al., 2018). Two case reports about concurrent pembrolizumab and pazopanib for primary hepatic angiosarcoma (PHA) (Qiao et al., 2018) and pembrolizumab plus sorafenib for HCC (Chen et al., 2017) did not observe any significant toxicity.

Regarding nivolumab, there were three studies examining concurrent nivolumab and pazopanib (Paoluzzi et al., 2016; Amin et al., 2018; Yu-Li Su, 2018), two for combining with sunitinib (Mahmoud et al., 2016; Amin et al., 2018), and one for combining with apatinib (Zhao et al., 2019), cabozantinib (Bhat et al., 2019) or sorafenib (Feng et al., 2017), respectively (**Table 2**). Zhao et al. observed grade 3 elevated aminotransferases in a patient with advanced liver carcinosarcoma treated with nivolumab plus apatinib (Zhao et al., 2019), while Bhat et al. did not observe severe trAEs in a patients treated with nivolumab plus cabozantinib (Bhat et al., 2019). In the phase 1 trial of Amin et al. 27 (81.8%) and 14 (70.0%) patients in arms nivolumab plus sunitinib and nivolumab plus pazopanib, respectively, experienced grade 3/4 trAEs, and 18 (55%) and 10 (50%) patients, respectively, experienced grade 3/4 irAEs (**Table 3**). The rate of arm nivolumab plus pazopanib was higher than that of arm nivolumab or pazopanib monotherapy in previous reports (Amin et al., 2018. Paoluzzi et al. reported 10 grade 3/4 trAEs in 4 (22.2%) patients receiving concomitant nivolumab and pazopanib, but no grade 3/4 trAEs occurred in nivolumab monotherapy group (Paoluzzi et al., 2016). Yu-Li Su et al. observed no toxicity after treatment of concurrent nivolumab and pazopanib in a patient with metastatic RCC (Yu-Li Su, 2018). Feng et al. analyzed nivolumab combined with sorafenib for advanced HCC in six patients and observed no severe toxicity (Feng et al., 2017), while Mahmoud et al. did not observe severe trAEs in a patient treated with nivolumab plus sunitinib (Mahmoud et al., 2016).

In summary, data on the toxicity of concurrent anti-PD-1 antibody and TKIs was conflicting. Some severe trAEs of the combination seemed to be largely related to TKIs, rather than a true irAE predominantly due to anti-PD-1 monotherapy. However, most studies were early phase clinical trials or case report, not randomized controlled studies with a large population, so the data is insufficient for conclusions.

#### Anti-PD-L1 (Avelumab, Durvalumab)

Two prospective studies (Choueiri et al., 2018; Motzer et al., 2019) evaluated concurrent use of avelumab and axitinib on advanced RCC. A total of 282 (57.7%) in 489 patients experienced grade 3–5 trAEs, of which the most frequent were diarrhea, hypertension, fatigue, palmar–plantar erythrodysesthesia syndrome, and changes of liver enzymes (**Table 3**). In addition, in the phase 1b trial of Choueiri et al. where 55 patients received avelumab plus axitinib, one patient developed a fatal treatment-related autoimmune myocarditis (Choueiri et al., 2018). In the prospective phase 3 trial of Motzer et al., three (0.7%) treatment-related deaths were attributed to sudden death, myocarditis, and necrotizing pancreatitis, respectively (Motzer et al., 2019) (**Table 3**). However, the trAEs observed with combination therapy were generally consistent with the known safety profiles of monotherapy. No new toxicities were reported.

Currently, only one phase 1 trial, treating a total of 14 patients with concurrent durvalumab and cediranib for several recurrent or metastatic solid tumors, was found (**Table 2**). Lee et al. observed 19 grade 3/4 trAEs occurred in 7 patients. In durvalumab plus intermittent cediranib, the severe AEs were only one grade 3 fatigue and one grade 4 hypertension. In contrast, daily cediranib with durvalumab was not well tolerated (**Table 3**) (Lee et al., 2017).

In summary, the very small number of patients treated with avelumab plus axitinib or durvalumab plus cediranib and lack of compared monotherapy group make it difficult to draw conclusions about their safety.

### Management of irAEs

In the included literatures, holding the ICI treatment was the first thing for managing grade 3/4 irAEs, and most of studies did not reduce the dose of ICIs, with an exception for one. In the case, a patient with HCC had a low-grade fever relating to remarkable tumor necrosis. Thus, to avoid tumor rupture, the schedule of pembrolizumab was changed to every 4 weeks (Chen et al., 2017). Besides, high-dose corticosteroids (including prednisone, methylprednisolone, and dexamethasone) were the first line for treating irAEs, and often effective in alleviating symptoms (**Table 3**). As some severe trAEs that were largely related to the addition of antiangiogenic agents, reducing or holding dose, as well as adjusting administration frequency of the antiangiogenic drugs, were the other common ways to deal with treatment-related toxicity (**Table 3**) (Carter et al., 2016; Lee et al., 2017; Amin et al., 2018; Atkins et al., 2018; Choueiri et al., 2018; Motzer et al., 2019; Rini et al., 2019a; Wilky et al., 2019). In addition, the rest of trAEs were managed with symptomatic treatment such as drugs or surgery (**Table 3**).

## Discussion

In this review, we demonstrated the risk of added toxicity of concurrent ICIs and antiangiogenic agents, but there are not abundant of data from multi-institutional randomized controlled trials (RCTs) to draw an exact conclusion. From the available data, bevacizumab and axitinib were the most commonly used antiangiogenic agents for concomitant treatment. For other antiangiogenic drugs, available safety information is primarily based on small, retrospective single institution experiences, and even case report. In terms of tumor types, the three most numerous studies on concurrent ICIs and antiangiogenic agents were RCC, non-squamous NSCLC, and CNS tumors (including glioblastoma) (**Tables 1** and **2**). However, the combination of the two types of therapies is indeed a research hotspot at present, with a huge amount of ongoing trials (**Table 4**).

**Table 4:** Parts of ongoing phase 2/3 clinical trials of ICIs combined with antiangiogenic agents.

ICIs	Antiangiogenic agents	Primary tumor	Status and end points	CliniclTrials.gov identifier
Nivolumab	Bevacizumab	Glioblastoma	Phase 2: recruiting (OS, ORR, DOR, and PFS)	NCT03452579
	Ramucirumab	Mesothelioma, malignant	Phase 2: recruiting (ORR, AEa, PFS, and OS)	NCT03502746
	Axitinib	Renal cell carcinoma	Phase 2: recruiting (AEs, ORR, DOR, PFS, OS, PD-L1 expression, and tumor infiltrating lymphocyte assessments, pharmacodynamic effect of study treatment including cytokines)	NCT03172754
	Cabozantinib	Renal cell carcinoma	Phase 3: recruiting (PFS, OS, ORR, AEs, SAEs)	NCT03141177
	Lenvatinib	Advanced hepatocellular carcinoma	Phase 2: recruiting (ORR, AEs, SAEs, TTP, PFS, OS, and translational research)	NCT03841201
	Regorafenib	Advanced and metastatic solid tumor	Phase 1/2: recruiting (RD, MTD, ORR, PFS, DCR, OS, and AEs)	NCT03406871
	Sunitinib	Soft tissue sarcoma, bone sarcoma	Phase 1/2: recruiting (PFSR, OS, ORR, immune response, tumor response, AEs, and clinical outcome)	NCT03277924
	Sorafenib	Hepatocellular carcinoma	Phase: recruiting (MTD, ORR, DOR, AEs, irAEs, OS, and PFS)	NCT03439891
(Nivolumab + ipilimumab)	Cabozantinib	Genitourinary tumors	Phase 2: recruiting (ORR, DOR, PFS, OS, CBR, AEs, and effects of treatment in patients with bone-only disease)	NCT03866382
	Nintedanib	Non-small-cell lung cancer metastatic	Phase 1/2: recruiting (MTD, ORR, DCR, OS, and PFS)	NCT03377023
SHR 1210 (anti-PD-1 mAb)	Apatinib	Gastric cancer and HCC	Phase 1/2: recruiting (OSR, tumor control rate, DCR, DOR, and AEs)	NCT02942329
Pembrolizumab	Bevacizumab	Colorectal cancer, metastatic cancer	Phase 2: recruiting (ORR, PFS, OS, and AEs)	NCT03475004
	Ramucirumab	Head and neck squamous cell carcinoma	Phase 1/2: recruiting (RP2D, ORR, AEs, DOR, PFS, OS, and changes in quality of life)	NCT03650764
	Apatinib	Advanced urothelial carcinoma, advanced MSI-H or dMMR solid tumors, advanced gastric or gastroesophageal junction (GEJ) adenocarcinoma	Phase 1/2: recruiting (DLTs, ORR, and PFS)	NCT03407976
(Pembrolizumab+ D-CIK)	Axitinib	Renal cancer metastatic	Phase 2: recruiting (ORR, PFS, OS, DOR, the quality of life, and AEs)	NCT03736330
	Anlotinib	Advanced solid tumor	Phase 2/3: recruiting (PFS, ORR, DCR, and OS)	NCT03975036
	Cabozantinib	Advanced metastatic melanoma	Phase 1/2: not yet recruiting (DLTs, ORR, DCR, PFS, and OS)	NCT03957551
	Lenvatinib	Thyroid gland carcinoma	Phase 2: recruiting (CR, AEs, PFS, OS, AEs, and biomarker levels)	NCT02973997
	Regorafenib	Metastatic colorectal cancer	Phase 1/2: not yet recruiting (DLTs, PFS, and OS)	NCT03657641
	Sunitinib	Thymic carcinoma	Phase 2: recruiting (ORR, AEs, OS, PFS, and PD-L1 expression)	NCT03463460
	Sorafenib	Hepatocellular carcinoma	Phase 1b/2: recruiting (ORR, OS. TTP, change in functional activity of effector T cells, and levels of immunosuppressive cell PFS)	NCT03211416
Atezolizumab (MPDL3280A)	Bevacizumab+chemotherapy	Ovarian cancer	Phase 3: recruiting (efficacy, TSST, OS, and AEs)	NCT02891824
	Ramucirumab	Non-small-cell lung cancer	Phase 2: recruiting (OS, CBR, and irAEs)	NCT03689855
	Cabozantinib	Hepatocellular carcinoma	Phase 3: recruiting (PFS and OS)	NCT03755791
Avelumab	Ramucirumab++paclitaxel	Gastroesophageal junction Adenocarcinoma/adenocarcinoma of the stomach	Phase 2: recruiting (OSR, OS, PFS, PFSR, DOR, ORR et al.)	NCT03966118
	Axitinib	Non-small-cell lung cancer; urothelial cancer	Phase 2: recruiting (ORR, TTR, tumor tissue biomarker status, ADA, DOR, PFS, Cmax of axitinib or avelumab, OS et al.)	NCT03472560
	Regorafenib	Metastatic solid tumors	Phase 1/2: recruiting (pharmacokinetics, RP2D, antitumor activity, MTD, DLT, toxicity, ORR, PFS, and blood biomarkers et al.)	NCT03475953
Durvalumab	Bevacizumab	Hepatocellular carcinoma	Phase 3: recruiting (RFS, OS, RFS24 h/36 h, TTR)	NCT03847428
	Pazopanib	Sarcoma	Phase 2: not yet recruiting (progression free rate: antitumor efficacy)	NCT03798106
MEDI4736 (anti-PD-L1 mAb)	Cediranib	Colorectal neoplasms; breast neoplasms	Phase 1/2: recruiting (RP2D and ORR)	NCT02484404

Usually, immune checkpoint blockade treatment is associated with multitude and atypical types of tumor responses and has specific toxicity profiles which are termed irAEs (Wolchok et al., 2009; Gordon et al., 2017). In general, within the first 3–4 months of treatment, 80% patients may experience irAEs (Chen et al., 2015; Michot et al., 2016). Because of the different functions of CTLA-4 and PD-1/PD-L1, the types and frequency of irAEs related to various checkpoint inhibitors were different (Michot et al., 2016). Anti-CTLA-4 antibodies mostly affect the skin (44%) and the gastrointestinal tract (35%), whereas the endocrine (6%) and hepatic (5%) systems are rarely affected (Boutros et al., 2016; Cousin and Italiano, 2016; Eggermont et al., 2016). The side effects of anti-PD-1/PD-L1 antibodies are less frequent and less severe than those of anti-CTLA-4 antibodies (Champiat et al., 2016; Puzanov et al., 2017). The main AEs of PD-1 and PD-L1 blocking agents are pneumonia, myalgia, hypothyroidism, arthralgia, and vitiligo (Boutros et al., 2016; Cousin and Italiano, 2016). In this review, the frequencies, types, and severities of irAEs that mentioned in most of studies were consistent with previous data for ICI treatment alone, and trAEs of combination regimen were largely consistent with the known safety profiles of each monotherapy. Besides, the data of included literatures suggested that some severe trAEs of the concurrent treatment were largely related to the addition of antiangiogenic agents, rather than a true irAEs caused by ICIs (Hodi et al., 2014; Atkins et al., 2018; Socinski et al., 2018; Reck et al., 2019). In addition, frequency of severe trAEs in ICI plus TKI groups was a little higher than that of ICIs plus mAbs groups, which may be explained by the multiple targets of TKIs. The toxicities consist of not only AEs related to the blockade of VEGR/VEGFR pathway but also AEs caused by additional targets inhibition (Chen and Cleck, 2009; Qin et al., 2019). For example, sunitinib (targeting VEGFR-1/2, PDGFR-α/β, Flt-3, and c-kit) is known to cause both neutropenia and thrombocytopenia as a result of VEGF inhibition and simultaneous inhibition of c-kit (Demetri et al., 2006; Chen and Cleck, 2009). Similarly, anemia and decrease of both platelet and neutrophil counts were observed in an included study of concurrent avelumab and axitinib (targeting VEGFR-1–3, PDGFR, and c-kit) (Matias et al., 2017; Rini et al., 2019a). Therefore, the selection of optimal components for combination therapy is worthy of further research.

In general, most irAEs are mild and manageable, although a few patients treated with ICIs develop severe irAEs (grade 3/4), even immune-related death (grade 5). Recommendations on the management of irAEs have been published as the guidelines in Europe and the United States (Puzanov et al., 2017; Brahmer et al., 2018; Haanen et al., 2018). Firstly, successful management of irAEs requires standardize grading based on the common terminology criteria for adverse events (CTCAE 4.0) grading. As for intervention, patients with grade 1 irAEs can continue immunotherapy, except for some neurologic, hematologic, and cardiac toxicities. Holding ICI treatment should be considered for most grade 2 irAEs until symptoms and/or laboratory values reduce to grade 1 or less and then treat them with locally or orally small doses of corticosteroids (0.5–1 mg/kg/d of prednisone or equivalent). For grade 3 irAEs, discontinuation of the ICI therapy and giving moderate to high-dose corticosteroids (prednisone 1–2 mg/kg/d or methylprednisolone i.v. 1 to 2 mg/kg/d) are recommended. Resuming treatment should be caution depending on the risk/benefit ratio. Regarding to life-threatening events (grade 4), hospitalization and high-dose corticosteroids (methylprednisolone i.v. 1-2 mg/kg/d) or other immunosuppressive measures (infliximab 5 mg/kg) are necessary. And ICI treatment should be permanently discontinued (Champiat et al., 2016; Puzanov et al., 2017; Brahmer et al., 2018). In the included studies, most of immune-related toxicities of the concurrent treatment were managed *via* holding ICI treatment and adding corticosteroids. Reducing dose or adjusting the administration frequency of the antiangiogenic drugs was also used to alleviate some symptoms of trAEs (**Table 3**). However, the information concerning the new advances and management of irAEs are limited.

Recently, irAEs were considered as therapy-induced loss of tolerance, similar to autoimmune disorders (Boutros et al., 2016; Postow et al., 2018; Pauken et al., 2019). Thus, the known risk factors for autoimmunity may also predict the risk of irAEs. Hoefsmit et al. 2019 searched for susceptible loci associated with various autoimmune diseases and pooled them in groups most likely to be associated with ICIs-induced irAEs (Hoefsmit et al., 2019), which may help to screening out patients with pre-existing subclinical autoimmune disorders or susceptibility to autoimmune diseases and guide physicians in a more refined and personal manner. Besides, depending on the degree of similarity between irAEs and autoimmune disorders, we can find reference in therapies developed for autoimmunity to manage irAEs (Pauken et al., 2019). For example, anti-TNF-α antibodies are usually used to treat steroid-refractory inflammatory bowel disease and could also alleviate ICIs-induced colitis (Dougan, 2017). Also, experts in autoimmune disorders should be involved in the care of cancer patients receiving ICIs. In addition, studies have found that gut microbiome is not only associated with the efficacy of immunotherapy but also with some specific irAEs, such as colitis (Osman and Luke, 2019). Thus, the ability to predict which patient has a high risk of developing ICI-induced colitis is very valuable to clinicians who have to weigh the potential risks and benefits of ICI therapy. Regarding to the combination of ICIs and antiangiogenic agents, the problem also includes the dose, optimal duration of treatment, and sequencing of each therapy. As is well known, anti-VEGF therapies have the window of normalization (Winkler et al., 2004; Huang et al., 2012), with the dose and duration time of antiangiogenic agents being the key modulating factors (Huang et al., 2012; Chaudhary et al., 2014). High dose or long duration time of antiangiogenic therapy are associated with aggressive ablation of the vasculature, leading to higher degree of hypoxia and immunosuppression (Huang et al., 2012; Allen et al., 2017). Thus, reducing the dose of antiangiogenic agents has been taken into account in the design of some clinical trials with the combination of ICIs (Fukumura et al., 2018). Besides, as vascular normalization can enhance delivery and distribution of ICIs in the tumor tissues, the low dose of ICIs may help to reduce the incidence and severity of irAEs (Fukumura et al., 2018). In addition, identification of predictive or prognostic markers is also expected to help screening suitable patients in order to prevent unnecessary side effects of combination therapy. Previous study found that expression level of PD-L1 was a predictive marker of the response to immunotherapy and also a negative prognostic marker in RCC patients receiving VEGF-targeted therapy (Shin et al., 2015). Angiopoietin 2 (ANG2), a vessel-destabilizing ligand of TIE2 and a critical regulator of blood vessel maturation, is a potential biomarker of resistance to anti-VEGF therapy (Bauerschlag et al., 2013; Jain, 2014; Labussiere et al., 2016). At the same time, evidence showing high serum level of ANG2 was inversely correlated with treatment response and prognosis of ICI treatment in metastatic melanoma patients (De Palma and Jain, 2017). Therefore, it is not so sensible to provide such a combined strategy for this kind of patients.

The current review has limitations that the number of available studies, especially RCTs, is insufficient. Even for some drugs, the data is lacking. These may partly due to the fact that many studies assessing the combination treatment of ICIs and antiangiogenic agents are still ongoing for this emerging area of research. Besides, the information about the new advances and management of irAEs in the included studies are limited. In addition, the review mainly focuses on three well known immune checkpoints, CTLA-4, PD-1, and PD-L1. However, identification of better biomarkers or therapeutic agents aimed at improving the clinical response in refractory patients and reducing irAEs is also necessary, which has led to the development of “next-generation” ICIs, such as T cell immunoglobulin mucin 3 (TIM-3), lymphocyte activation gene 3 (LAG-3), T-cell immunoreceptor with immunoglobulin and ITIM domains (TIGIT), indoleamine-2,3-dioxygenase 1 (IDO1), and so on (Mazzarella et al., 2019). Hundreds of registered past and ongoing clinical trials investigate the mechanism and efficiency of “next-generation” ICIs either as monotherapy or combining with other ICIs (Mazzarella et al., 2019; Tundo et al., 2019). Therefore, updated information is still required in the future.

## Conclusion

In summary, concurrent ICIs and antiangiogenic agents show potential treatment-related toxicity. Further research is required to compare the efficacy and safety of the combined regimen and the corresponding monotherapy. It is also necessary to explore dose, duration, and sequencing schedule of drugs, as well as identify predictive or prognostic biomarkers.

## Author Contributions

LG, XY and HZ conceived and designed the study. LG and XY screened, extracted the data, and wrote the manuscript. HZ and CY contributed to the revise of the manuscript.

## Funding

This research was supported by the Post-Doctor Research Project, West China Hospital, Sichuan University (NO. 2018HXBH032) and Sichuan Science and Technology Program (NO. 2019YFS0109).

## Conflict of Interest

The authors declare that the research was conducted in the absence of any commercial or financial relationships that could be construed as a potential conflict of interest.
